# Supplementary material for: A Solar cycle correlation of coronal element abundances in Sun-as-a-star observations
Source: Nat Commun. 2017 Aug 3;8:183. doi: 10.1038/s41467-017-00328-7 (PMC5543058; doi:10.1038/s41467-017-00328-7)
Supplement: Supplementary file 1 — Supplementary Information [file 41467_2017_328_MOESM1_ESM.pdf]

File name: Supplementary Information
Description: Supplementary Figures

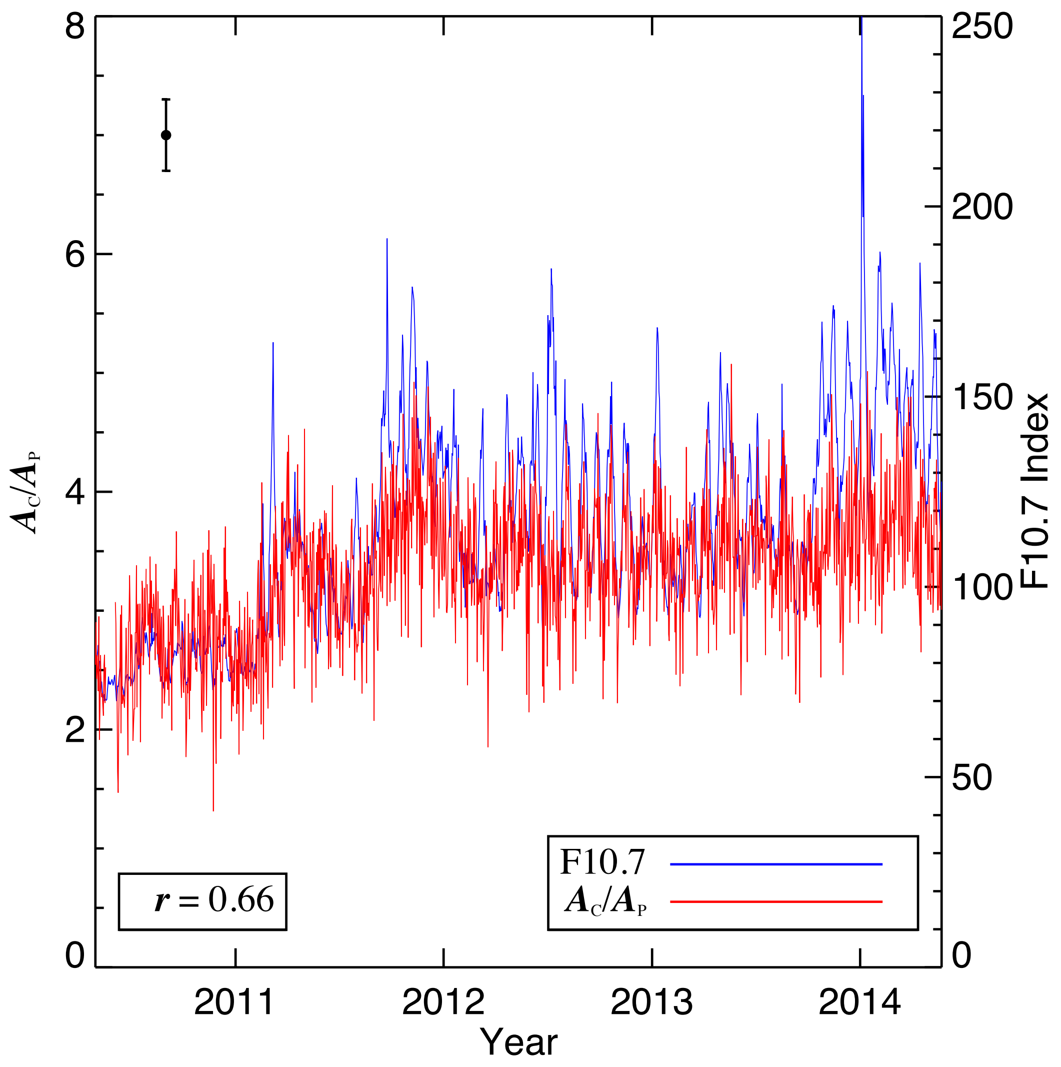

Supplementary Figure 1: Daily running averaged composition and F10.7 cm data.

The evolution of the daily running averaged ratio of the coronal to photospheric composition in the Sun's corona between April 2010 and May 2014 (red) and the evolution of the daily running averaged F10.7 cm radio flux during the same period. We show the correlation coefficient in the legend. We also show the uncertainty in the composition measurements with an error bar. This is the dispersion (standard deviation) in A_c/A_p computed from variations in the best fits to the irradiance data from a large number of random trials (see Methods for more details). The slightly lower correlation compared to Figure 2 may be because detailed daily variations due to activity, such as flares, are smoothed out in the Carrington 27-day running average data.

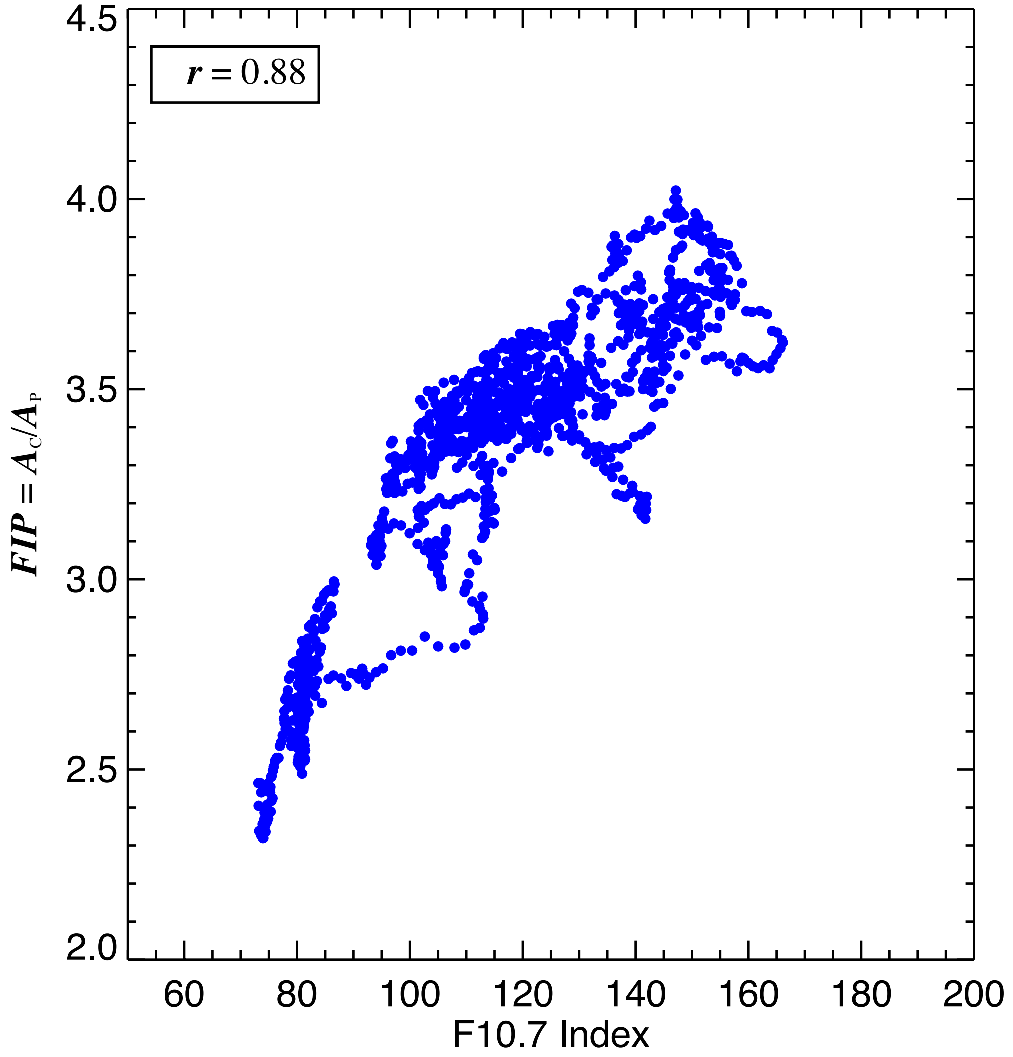

Supplementary Figure 2: Correlation plot of the composition and F10.7 cm data.

Correlation between the 27-day Carrington rotation running averaged A_c/A_p ratio and the F10.7 cm radio flux for April 2010 to May 2014. We show the correlation coefficient in the legend.

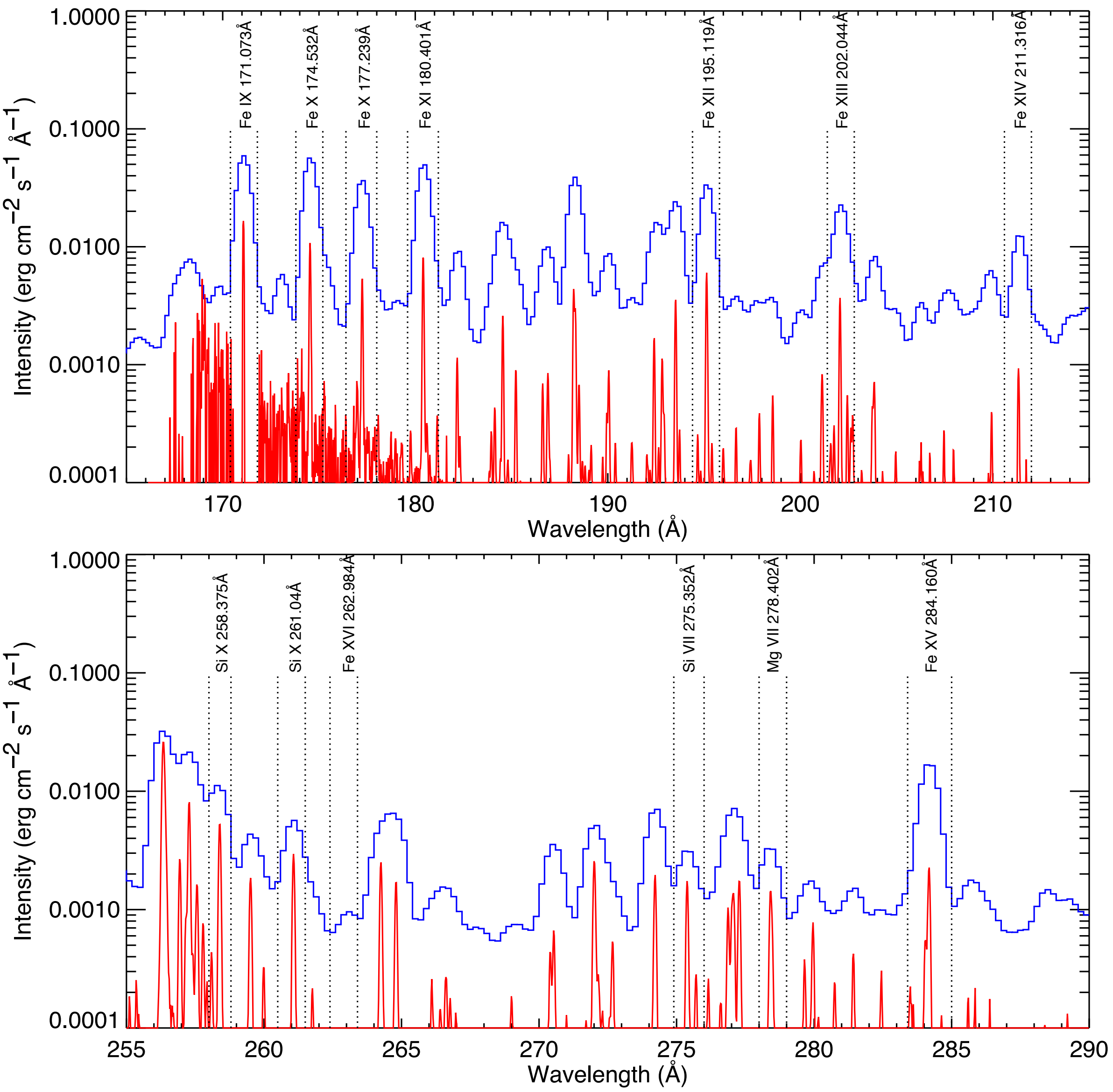

Supplementary Figure 3: Example of the EVE spectra.

Example daily averaged EVE (Extreme ultraviolet Variability Experiment) spectra taken on 13th May 2010 (blue histogram). We have labelled the spectral lines used in the DEM (Differential Emission Measure) analysis, and the vertical dotted lines show the wavelength limits that were used to extract the intensities. We show a much higher spectral resolution quiet Sun EIS (Extreme-ultraviolet Imaging Spectrometer) spectrum for comparison (red line). The EIS spectrum was taken on 30th January 2007 using a much smaller field-of-view of 128 by 128 arcseconds.

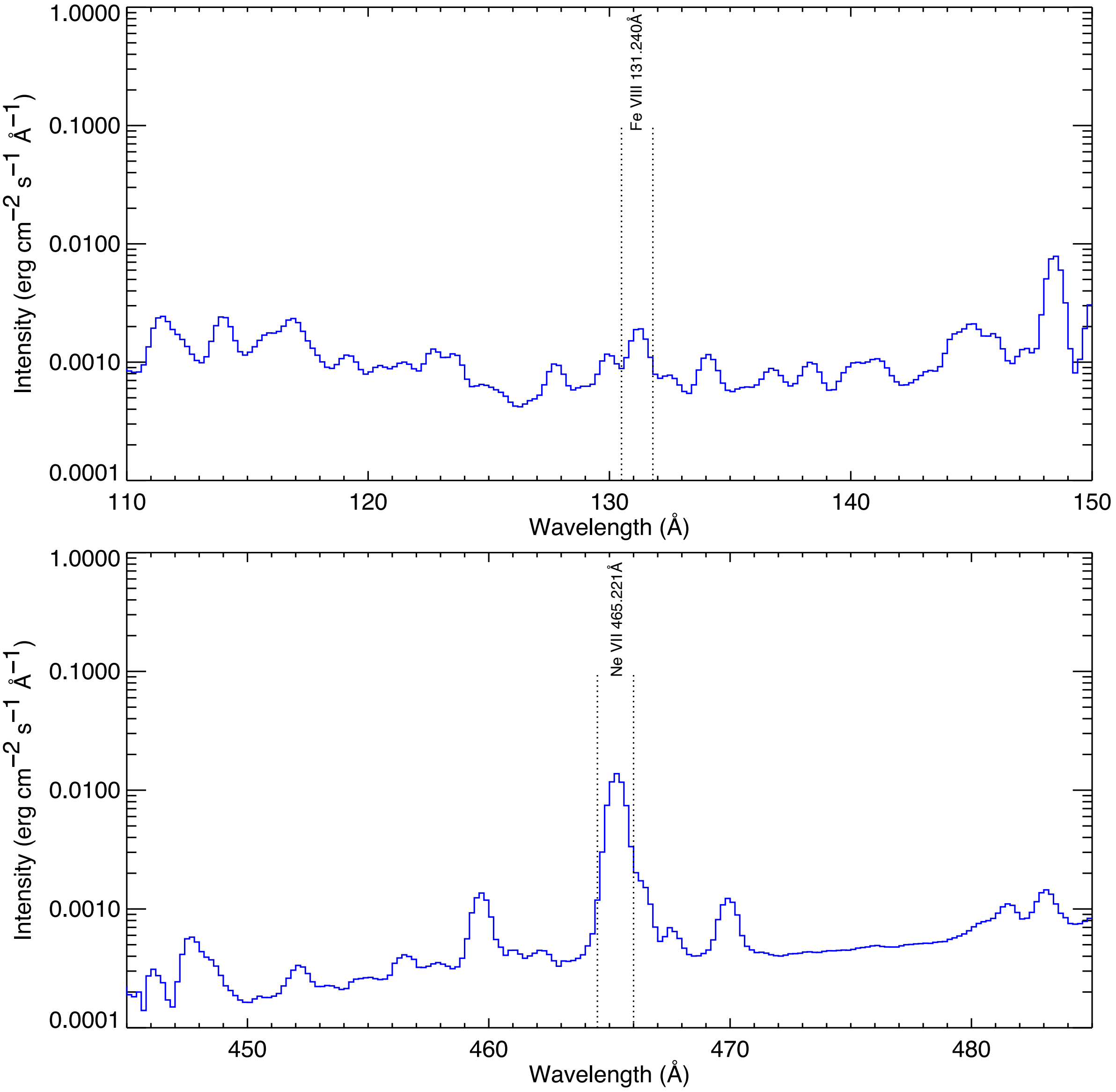

Supplementary Figure 4: Example of the EVE spectra.

An example of the daily averaged EVE (Extreme ultraviolet Variability Experiment) spectra taken on 13th May 2010 (blue histogram) showing the wavelength intervals of the Fe VIII 131.24 Å line that was included to better constrain the lower temperatures, and the Ne VII 465.221 Å line that was used to measure the coronal to photospheric composition ratio.

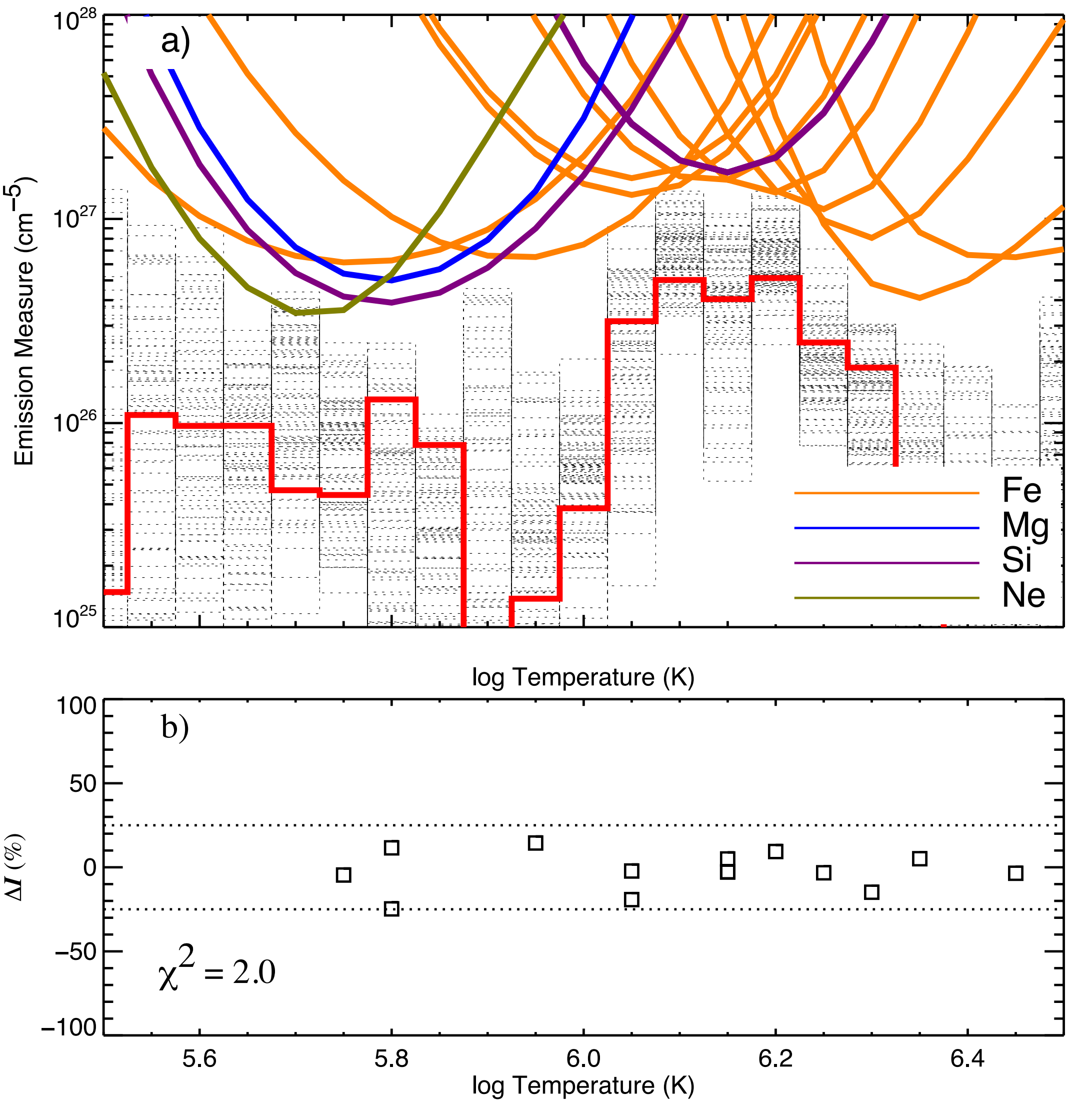

Supplementary Figure 5: Example of the coronal temperature distribution.

a, Example DEM (Differential Emission Measure) solution for the daily averaged spectrum taken on 13th May 2010. The dotted grey lines show the Monte Carlo simulations and the red line shows the best-fit solution. The colored lines are emission measure loci curves for all the spectral lines and show the upper limit constraints on the DEM. The Ne VII curve is the model solution. **b**, Differences between the observed and DEM calculated intensities expressed as a percentage of the observed intensity. The differences are all less than 25% and the resultant chi squared value for the fit is low (see legend).

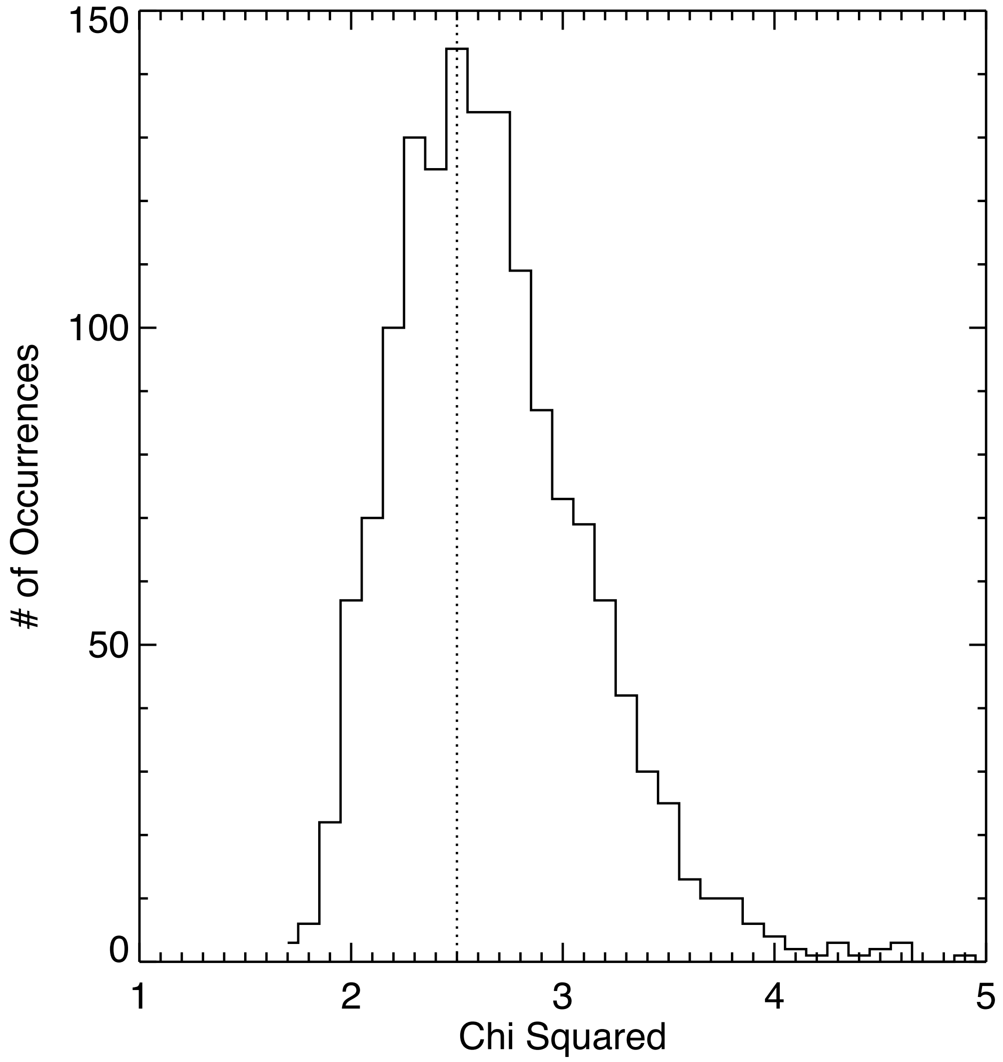

Supplementary Figure 6: Chi squared values for the complete dataset.

Histogram of chi squared values for each best-fit DEM (Differential Emission Measure) solution to all the individual daily averaged spectra in the complete 4-year dataset. The results cover the time period from April 2010 to May 2014.

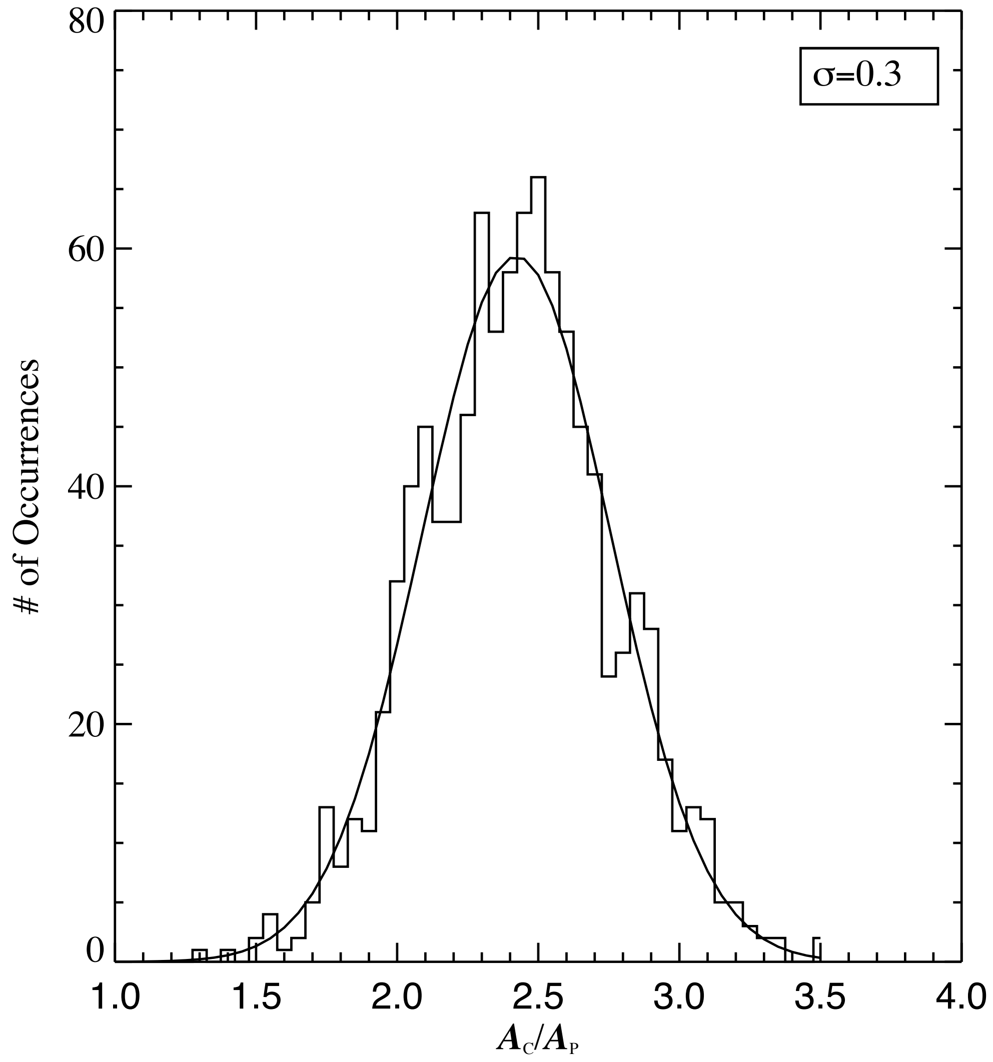

Supplementary Figure 7: Example of the dispersion in the computed composition ratios. Histogram of coronal to photospheric composition ratio for 1000 independent trial simulations for the 13th May 2010 dataset. Each of the 1000 measurements are computed from the best fit DEM (Differential Emission Measure) solution obtained from a sample of 100 MCMC (Markov-Chain Monte Carlo) simulations that adjust the DEM to fit the observed irradiances. We show the standard deviation, indicating the dispersion of the measurements, in the legend.
